# Open Face Crown vs. Contour Filling or Richmond Crown for Incisors

**Published:** 1899-10

**Authors:** H. T. King

**Affiliations:** Fremont, Nebraska.


ARTICLE X.

### OPEN FACE CROWN VS. CONTOUR FILLING OR RICHMOND CROWN FOR INCISORS.

H. T. KING, D. D. S., FREMONT, NEBRASKA.

How would I restore the tooth a little too far gone to fill, and the loss of structure does not necessitate a crown? In my title, I have designated this as an open face crown; it is hardly that, for as shell crowns are usually made, as little gold and as much cement as possible is used, while in this case, as much gold as is necessary to make contour, and as little cement as possible is used. It is hardly an inlay, either, although the inlay principle is used in making it. I proceed as follows:

First, trim away all enamel which is frail, but leave much more of the labial plate than would be admissible for a foil filling. With disks, remove all the enamel from the proximal portion of the cervix on both sides, giving a flat, highly polished surface. Remove a portion, at least, of enamel from lingual aspect. Shorten tooth a very little, beveling from both sides. Cut out decay, and if this leaves the cavities so deep or irregular that impression can not be taken, fill the deep parts with gutta percha. The work can all be nicely done in the mouth, but I find that time is saved and the patient relieved, by taking a plaster impression and making a fusible metal cast. A perfect reproduction of the tooth in metal can be quickly obtained by slipping into the proximal space on either side, a piece of cardboard just thick enough to fit, letting it press the gum back a little from the interproximal space.

If I wish a pin or dowel to extend into the pulp canal or deep portion of cavity for anchorage, a piece of round wood toothpick is put in place, and comes away with cardboard in impression. When the impression is fitted with Melotte's metal, I have a more or less perfect model, to which my gold is approximately fitted.

Take a generous piece of 24k. gold, 32 to 36 gauge, and by pressing, burnishing and trimming over all the lingual and proximating surfaces of tooth, the gold on the proximal sides cut off just even with cutting edges, that covering the lingual side extending so as to fold over and fit to the shortened occlusal surface.

My way is to first fit to one proximal surface, forcing gold into more depressed portion, with a piece of erasing rubber under instrument. Slip from model and flow enough 22 k. solder to stiffen it. Replace on model, drive piece of soft wood between adjoining tooth and gold that have been melted on, thus holding firmly in place while I burnish and fit to the whole lingual surface; remove and flow on gold. Replace, fasten in place, and fit gold to other cavity and proximal surface. The gold being thin and pure, all this can be quickly done.

The solder is to be laid on in small pieces, a little at a time, and heat enough given to barely melt it, for, until it is fitted to tooth in mouth, no solder is to be allowed to flow onto the part that it is to be fitted over end of tooth and edges of labial plate of enamel. This is important, for upon the close adaptation of gold to tooth at exposed point depends the good appearance of the finished work, and, in a great measure, its durability as well. I have an idea that the best way to add piece by piece of solder is by the use of the mouth blowpipe. Possibly that is because I enjoy the use of that instrument, and have become somewhat of an expert from having done all my crown and bridge work in that way. Others may get as good results from a mechanical blowpipe and bellows, but I have no use for them, except to melt a large batch of gold.

Our piece of work is now fitted to the model and made rigid enough to handle without fear of bending. We remove from model to tooth in the mouth and burnish to the enamel. Remove very carefully, paint the inside with whiting or finely-ground asbestos and invest; sufficient gold is then added to make necessary contour. You are all workers in gold and have your own way of doing this, so I need not dwell upon it. A perfect polish out of the mouth is to be given to all parts except the labial face; a rough polish will do there, as this part is to receive its final polish in the mouth after cement has hardened.

If the work has been neatly done, cement will not show. and you have, to all appearance, a contour gold filling with less gold showing, however, than if built up and with an occlusal surface made of hard gold, that may with safety be brought to as thin a cutting edge as desired.

I have said nothing of anchorage, and some may be asking the question, "Will it stay ?" The tooth having gole on all sides except labial, no ordinary force can dislodge it when applied in any direction, unless down or in —the two directions not necessary to particularly guard on the upper jaw. As a rule, I depend upon the fitting of gold into the two proximal cavities and turning over the beveled occlusal edge, or, if not necessary to turn the end and have gold show, fit into groove cut through occlusal surface. This is but slight anchorage, but sufficient to prevent the crown from tipping in, unless it first comes down a little.

The method I present for the restoration to form and usefulness of badly decayed incisor teeth is not universal in its application, but in the last six years I have found many places where it seemed to me the best method, and it has given great satisfaction, both to myself and patient. I have used it oftenest on frail lateral incisors of the upper jaw, but in a few cases have found it just the thing for lower incisors so badly gone that I have doubted my ability to restore with gold fillings, and did not consider them good subjects for porcelain crowns. Have also had some pleasing results from supplying the principle to bicuspids, when I have found the buccal enamel and cusp in good condition, yet the tooth demanding a crown of some kind. You can give such a tooth the appearance of having a contour filling, with but little more work than making a shell crown, and the finished work will be much less conspicuous. For a bicuspid, a good strong pin should extend from gold on occlusal surface well into pulp canal, as strong anchorage is demanded.—"Review."

